# Cutaneous candidiasis in Tehran-Iran: from epidemiology to multilocus sequence types, virulence factors and antifungal susceptibility of etiologic *Candida* species

**Published:** 2019-08

**Authors:** Golnar Sadeghi, Mina Ebrahimi-Rad, Masoomeh Shams-Ghahfarokhi, Zahra Jahanshiri, Esmat Mirabzadeh Ardakani, Ali Eslamifar, Seyed Fazlollah Mousavi, Mehdi Razzaghi-Abyaneh

**Affiliations:** 1 Department of Medical Mycology, Pasteur Institute of Iran, Tehran, Iran; 2 Department of Biochemistry, Pasteur Institute of Iran, Tehran, Iran; 3 Department of Medical Mycology, School of Medical Sciences, Tarbiat Modares University, Tehran, Iran; 4 Biotechnology Research Center, Pasteur Institute of Iran, Tehran, Iran; 5 Department of Clinical Research, Pasteur Institute of Iran, Tehran, Iran; 6 Department of Microbiology, Pasteur Institute of Iran, Tehran, Iran

**Keywords:** *Candida* species, Candidiasis, Molecular epidemiology, Virulence factors, Antifungal susceptibility, Multilocus sequence typing

## Abstract

**Background and Objectives:**

Cutaneous candidiasis is a multipicture fungal infection caused by members of the genus *Candida* which is considered as a public health problem all over the world with urgency of effective treatment and control. This study was performed to analyze the clinical epidemiology and molecular aspects of cutaneous candidiasis in Tehran-Iran in relation to antifungal susceptibility and virulence factors of etiologic *Candida* species.

**Materials and Methods:**

*Candida* species were isolated from skin (27.3%) and nail scrapings (72.7%) of suspected patients and identified by ITS sequencing. Phylogeny of the isolates was evaluated using multilocus sequence typing (MLST) and antifungal susceptibility and virulence factors of the isolates were determined in relation to clinical presentation.

**Results:**

*Candida albicans* was the most prevalent species (39.8%), followed by *C. parapsilosis* (32.9%), *C. orthopsilosis* (10.4%), *C. tropicalis* (7.9%), *C. glabrata* and *C. guilliermondii*, each (4.5%). Molecular typing of 35 *C. albicans* isolates by MLST revealed 28 novel sequence types with 11 singletons with 80.0% new diploid sequence types (DSTs). Majority of the isolates were susceptible to amphotericin B (91.5%), followed by posaconazole (90.3%), fluconazole (84.3%), itraconazole (74.1%), caspofungin (53.6%), and voriconazole (26.8%). Biofilm formation, yeast-to-hyphae transformation and phospholipase activity were reported species-dependent.

**Conclusion:**

Our results demonstrated clinical epidemiology of various *Candida* species from cutaneous candidiasis distributed in new molecular types with increasing importance of drug resistant of non-*albicans Candida* species. Our results showed that drug susceptibility and genetic variability of *Candida* species may be attributed to their clinical features and source of isolation.

## INTRODUCTION

Although groups of fungi are part of the commensal skin microbiota, various species are also pathogenic. It has been estimated that 20–25% of the world population is affected by fungal skin infections. *Candida* species are the second factors of dermatomycoses (1). Cutaneous *Candida* infections may occur in patients with HIV/AIDS, cancer, receiving chemotherapy, antibiotics, steroid therapy and solid organ transplantation (2).

During recent years, in different geographical parts, due to patients predisposing conditions and the types of antifungal agents received, changes in species distribution of *Candida* have also happened. Changes in species distribution may impact treatment recommendations because of differences in susceptibility to these antifungals among these species (3). Previous exposure to antifungals is clearly associated with a shift in species distribution and MICs of available antifungal agents (4).

The previous data clearly showed that the selection and optimization of an antifungal regimen in treating *Candida* infections are based on multiple factors such as knowledge of local epidemiological data to guide experiential therapy as well as both species identification and antifungal susceptibility testing (5, 6).

Although *Candida albicans* is the most prevalent species involved in cutaneous candidiasis, there has been an increase in the number of non-*Candida albicans* species (NCA), globally. This fact is essentially due to the rise in antimicrobial resistance and the restricted number of competent antifungal drugs, which still have many side effects (7).

The pathogenicity of *Candida* species is attributed to certain factors, such as the ability to evade host defences by filamentous forms, biofilm formation capacity, and the production of tissue damaging hydrolytic enzymes such as phospholipase (8).

Typing strains within a microbial species on the basis of DNA sequences at multiple loci has greatly advanced study of the epidemiology and evolutionary phylogenetic of many fungal pathogens (7). The ability to discriminate between fungal strain types has been a topic of great importance to epidemiologists. The MLST approach offers a number of advantages for studying the geographic distribution, prevalence diversity, genetic basis of pathogenicity, and drug resistance of *Candida* species (9, 10).

The aim of the present study was to evaluate Molecular epidemiology, phylogeny and distribution of *Candida*-related infections with special reference to antifungal susceptibility of identified *Candida* species to current antifungals and their ability to produce virulence factors which facilitate the fungal pathogenicity.

## MATERIALS AND METHODS

### Clinical samples and species identification.

During our study, 120 cases suspected to cutaneous candidiasis who referred to our Department of Medical Mycology, Pasteur Institute of Iran were studied. Clinical samples were prepared and examined directly according to the standard protocols and inoculated on the Sabouraud dextrose agar with Chloramphenicol (SC) (Merck, Germany) and incubated at 28°C for one week and 16% were positive. Confirmation and species identification of isolates was done by combination of micromorphological features (germ tube test, morphology on corn meal agar), chromogenic assay on CHROMagar *Candida* (CHROMagar Candida, France), biochemical tests such as API ID32C system (bioMèrieux, Marcy l’Etoile, France) and ITS sequencing according to the manufacturer’s instructions. Standard strains of *C. krusei* ATCC (American type culture collection) 6258 was used for quality control.

### Molecular identification of *Candida* species: PCR amplification.

For DNA extraction, fresh colonies were collected upon culturing the isolates on Sabouraud dextrose agar (SDA) for 48 h at 37°C. Genomic DNA was extracted using the Molecular Biology kit (Bio Basic Inc, Canada) according to the manufacturer’s instructions and stored at −20°C until used. The molecular identification was performed by sequencing the internal transcribed spacer (ITS) region with ITS1 (5′-TCCGTAGGTGAACCTGCGG-3′) and ITS4 (5′-TCCTCCGCTTATTGATATGC-3′) primers as previously described by Bitar et al. (11).

### DNA sequencing.

**Fig. 1:**
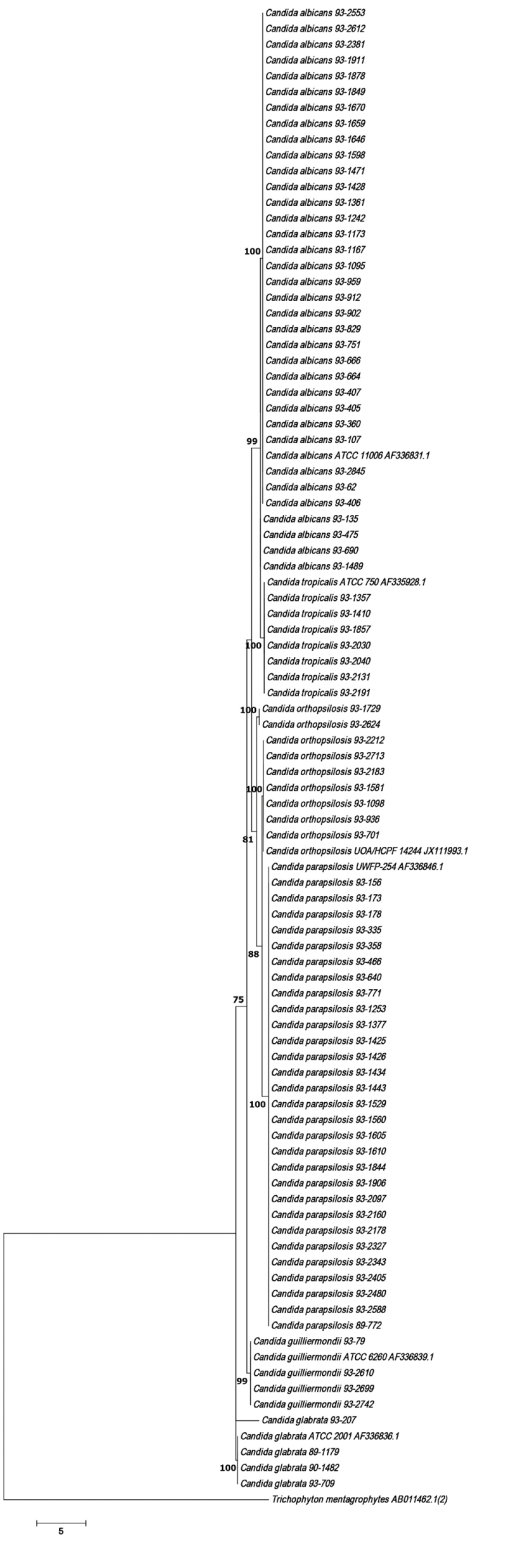
ITS Maximum Likelihood tree showing the phylogenetic relationship among *Candida* isolates. Bootstrap percentages from 1000 replicates are shown in each node.

The amplicons were purified and the subsequent sequencing reaction was performed using Big Dye Terminator version 3.1 Cycle Sequencing Kit (Applied Biosystems) on an ABI PRISM 377 Genetic Analyzer (Applied Biosystems). Nucleotide sequences were edited and defined by alignment of forward and reverse sequences using Sequencher 5.4.6 and MEGA version 7.0. To perform the phylogenetic analysis, ITS sequences from type strains were downloaded from GenBank (http://www.ncbi.nlm.nih.gov/genbank/) after BLASTn searches (http://www.ncbi.nlm.nih.gov/). Phylogenic analyses were conducted by maximum likelihood algorithms. The robustness of the phylogeny was evaluated using 1000 bootstrap replications (Fig. 1) (12).

### *In vitro* antifungal susceptibility testing.

Antifungal susceptibility testing was performed by the reference broth microdilution method guidelines and Categorical susceptibility was assessed using the CLSI M27-S4 breakpoints and epidemiological cutoff values (ECVs) where applicable (13–15).

### Virulence factors.

The *in vitro* biofilm formation of *Candida* isolates was determined as described by Zago et al. (16). The metabolic activity of the biofilm was measured by crystal violet (CV) (1% v/v) assay after 48 h of incubation. Based on the average optical densities (ODs) observed, the isolates were classified into three groups showing: (i) low biofilm-forming (OD less than 0.12); (ii) medium biofilm forming (OD of 0.12–0.20), or (iii) high biofilm-forming (OD higher than 0.20). Phospholipase activity was evaluated by method as mentioned by Tsang et al. and Galan-Ladero et al. (17, 18). Suspension with 10^8^ cell/ml was dropped onto each test medium. After 48 h of incubation at 37°C, the diameters of the colonies (a) and the diameters of the precipitation zone around the colonies (b) were measured. The production of the enzyme was designated as Pz = a/b. The following ranges of activity according to Pz index were established: high, Pz = 0.40; medium, Pz = 0.41–0.60; low, Pz = 0.61–0.80; very low, Pz = 0.81–0.99; none, Pz = 1. The Pz index is a reproducible semi-quantitative technique used widely. Pseudohyphae formation was defined as a cell bearing a rounded outgrowth with a length greater than or equal to the diameter of the parent cell, with a constriction at the base. The percentage of cells in pseudohyphae form, against blastopores after 2 h of cell growth in a liquid medium containing equal volume of RPMI 1640 (Sigma) and fetal bovine serum (GIBCO, USA) was determined by microscopy counting as described by Negri et al. (19).

### Multilocus sequence typing (MLST).

For DNA extraction, fresh colonies were collected upon culturing the isolates on Sabouraud dextrose agar (SDA) for 48 h at 37°C. Genomic DNA was extracted using the Molecular Biology kit (Bio Basic Inc, Canada) according to the manufacturer’s instructions and stored at −20°C until used. The seven used consensus gene sets in the MLST analysis were included; AAT1a, ACC1, ADP1, PMI1b, SYA1, VPS13, and ZWF1b. The gene products, chrosomal localization, primer sequences and number of bases analyzed have been presented in detail earlier (20) as well as the central internet database for global MLST data (http://calbicans.mlst.net/). Fragment amplification by PCR was carried out in a 25 μL reaction volume containing 1 μg of genomic DNA. The amplification was performed using the protocol as described by Gammelsrud et al. (21). The amplicons were purified and the subsequent sequencing reaction was performed using Big Dye Terminator version 3.1 Cycle Sequencing Kit (Applied Biosystems) on an ABI PRISM 377 Genetic Analyzer (Applied Biosystems). The two overlapping strands were aligned using the Sequencher 5.4.6 software (Gene codes Corporation, Ann Arbor, MI, USA) and contigs were constructed. All contigs were analyzed peak-by-peak, and heterozygosity was identified by the presence of two peaks at the same polymorphic loci on both strands.

Allele numbers and diploid sequence types (DSTs) were found in the central MLST database (http://calbicans.mlst.net/). Chromatograms for the isolates with new alleles and/or new DSTs were sent to the central MLST database where new numbers were assigned. eBURST analysis was performed using the eBURST software 3.0 (22). The DSTs were compared to all DSTs included in the consensus MLST database as of July 2018, and divided into eBURST clonal complexes (CC). CC were named with the reference DSTs in concordance to previously described major *C. albicans* clades (23, 24).

### Statistical analysis.

Results obtained were analyzed using the under Windows SPSS program Version 16.0. One- way ANOVA with the Tukey post-hoc test was used. All tests were performed with a confidence level of 95%.

## RESULTS

### Isolation and conventional identification of *Candida* species.

Out of 1588 examined patients, 545 cases were showed dermatomycoses. Among them, 110 cases were positive for cutaneous candidiasis. Of which, 88 sample had growth on culture media. *Candida* isolates collected from cutaneous clinical samples, *C. albicans* (39.8%) was the most prevalent species followed by *C. parapsilosis* (32.9%), *C. orthopsilosis* (10.4%), *C. tropicalis* (7.9%) and *C. glabrata* and *C. guilliermondii*, each (4.5%). The mean age of patients was 50.8 years (range: 2 to 88 years), 64.8% were female. The epidemiological characteristics associated with cutaneous candidiasis were shown in Table 1. Among cutaneous lesions, the most frequent affected regions were the nails (64 cases; 72.7%), whereas the least affected site was the glabrous skin region (2 cases; 2.3%). The prevalence of onychomycosis caused by *Candida* species was 50 cases (78%) in women and 14 cases (22%) in men. Clinical files were available for 53 (69.2%) of patients and type 2 diabetes were the most prevalent predisposing factor. Distribution of *Candida* species by anatomical sites of infection were shown in Table 2. The most prevalent cause of nail infections was *C. parapsilosis*, whereas in *C. albicans* was the main etiologic agent of hands infection.

**Table 1.** Epidemiological characteristics associated with cutaneous candidiasis

**Parameter**	**Candidiasis No. (%)**
**Anatomic sites**	
Fingernails	49 (55.7)
Toenails	15 (17.0)
Hands	13 (14.8)
Feet	6 (6.8)
Groins	3 (3.4)
Glabrous skin	2 (2.3)
**Species**	
*C. albicans*	35 (39.8)
*C. parapsilosis*	29 (32.9)
*C orthopsilosis*	9 (10.4)
*C. tropicalis*	7 (7.9)
*C. glabrata*	4 (4.5)
*C. guilliermondii*	4 (4.5)
**Gender**	
Female	57 (64.8)
Male	31 (35.2)
**Age**	
0–29	16 (18.2)
30–59	47 (53.4)
60–89	25 (28.4)
**Predisposing factors**	
Diabetes	25 (28.4)
immunosuppressive treatment	15 (17.0)
rheumatoid arthritis	10 (11.4)
Other factors	3 (3.4)
None	35 (39.8)
**Total**	88 (100)

**Table 2.** Distribution of *Candida* species among clinical specimens.

**Species**	**Anatomic sites**					

	**Fingernails**	**Toenails**	**Hands**	**Feet**	**Groins**	**Glabrous skin**
*C. albicans*	9 (18.3)	5 (33.3)	12 (92.3)	5 (83.3)	3 (100)	1 (50.0)
*C. parapsilosis*	19 (38.8)	8 (53.3)	1 (7.7)	1 (16.7)	0 (0)	0 (0)
*C. orthopsilosis*	9 (18.3)	0 (0)	0 (0)	0 (0)	0 (0)	0 (0)
*C. tropicalis*	7 (14.3)	0 (0)	0 (0)	0 (0)	0 (0)	0 (0)
*C. glabrata*	3 (6.1)	0 (0)	0 (0)	0 (0)	0 (0)	1 (50.0)
*C. guilliermondii*	2 (4.2)	2 (13.4)	0 (0)	0 (0)	0 (0)	0 (0)
Total	49 (100)	15 (100)	13 (100)	6 (100)	3 (100)	2 (100)

Values are given as n (%).

### Molecular identification.

Identification of species by conventional methods was confirmed by typing of ITS genes in most cases and all of the *C. orthopsilosis* isolates were detected by this method, exclusively. No *C. metapsilosis* strains were found. The evolutionary relationships of taxa were presented in Fig. 1. The evolutionary history was inferred using the Maximum Liklihood method.

### Antifungal susceptibility testing.

The range of susceptibility according to M27-S4 and ECVs, MIC ranges and geometric means of *Candida* species for the six antifungal agents consistent with CLSI standard documents were summarized in Table 3. Majority of the isolates were susceptible to amphotericin B (91.5%), followed by posaconazole (90.3%), fluconazole (84.3%), itraconazole (74.1%), caspofungin (53.6%), and voriconazole (26.8%).

**Table 3.** *In vitro* susceptibility testing of *Candida* species to seven antifungal agents determined by CLSI broth microdilution method

**Species (No.)**	**Antifungal drug**	**MIC Range (μg/mL)**	**Susceptibility (%)**					**Geometric mean**
			**CBP**			**ECV**		**MIC**

			**S**	**R**	**SDD**	**WT (S)**	**Non-WT (R)**	
*C. albicans* (35)	FCZ	0.012–2	100	0	0	82.0	18.0	0.2928
	VOR	0.062–8	71.4	8.6	20.0	72.2	27.8	0.2264
	ITR	0.062–4	51.4	14.3	34.3	51.4	48.6	0.2133
	POS	0.031–16	-	-	-	88.6	11.4	0.0509
	CAS	0.031–4	48.6	37.1	14.3	22.9	77.1	0.4018
	AmB	0.031–2	-	-	-	100	0	0.3781
	KCZ	0.031–4	-	-	-	-	-	0.0905
*C. parapsilosis* (29)	FCZ	0.12–2	100	0	0	100	0	0.0704
	VOR	0.062–2	24.1	13.8	62.1	24.1	75.9	0.3171
	ITR	0.062–2	-	-	-	93.1	6.9	0.2325
	POS	0.031–0.062	-	-	-	100	0	0.0499
	CAS	0.062–8	96.6	3.4	0	89.7	10.3	0.5500
	AmB	0.12–4	-	-	-	96.0	4.0	0.3869
	KCZ	0.031–1	-	-	-	-	-	0.1027
*C. orthopsilosis* (9)	FCZ	0.25–4	-	-	-	88.0	12.0	0.9258
	VOR	0.25–2	-	-	-	0	100	0.7348
	ITR	0.12–2	-	-	-	-	-	0.4286
	POS	0.031–0.12	-	-	-	100	0	0.0725
	CAS	0.031–2	-	-	-	55.6	44.4	0.6293
	AmB	0.12–2	-	-	-	-	-	0.7058
	KCZ	0.062–0.5	-	-	-	-	-	0.1455
*C. glabrata* (4)	FCZ	0.25–64	0	25.0	75.0	75.0	25.0	2.16653
	VOR	2–8	-	-	-	0	100	2
	ITR	0.5–1	-	-	-	100	0	0.7262
	POS	0.031–0.12	-	-	-	100	0	0.1331
	CAS	0.031–1	25.0	75.0	0	25.0	75.0	0.1384
	AmB	0.031–2	-	-	-	80.0	20.0	0.4494
	KCZ	0.12–4	-	-	-	-	-	0.4868
*C. guilliermondii* (4)	FCZ	0.062–8	-	-	-	75.0	25.0	0.7071
	VOR	0.062–4	-	-	-	50.0	50.0	0.4204
	ITR	0.062–0.5	-	-	-	100	0	0.2973
	POS	0.12–16	-	-	-	75.0	25.0	0.4204
	CAS	0.25–1	100	0	0	100	0	0.3535
	AmB	0.12–1	-	-	-	100	0	0.2973
	KCZ	0.031–0.5	-	-	-	-	-	0.1487
*C. tropicalis* (7)	FCZ	0.25–16	85.7	14.3	0	85.7	14.3	0.7430
	VOR	0.062–1	14.3	28.6	57.1	14.3	85.7	0.4102
	ITR	0.062–0.5	-	-	-	100	0	0.2050
	POS	0.031–0.25	-	-	-	85.7	14.3	0.0690
	CAS	0.12–4	57.1	28.6	14.3	28.6	71.4	0.4102
	AmB	0.062–4	-	-	-	70.0	30.0	0.6095
	KCZ	0.062–0.25	-	-	-	-	-	0.1250

MIC: Minimum Inhibitory Concentration, (-) breakpoints not provided by CLSI documents, FCZ: Fluconazole, VOR: Voriconazole, ITR: Itraconazole, POS: Posaconazole, CAS: Caspofungin, AmB: Amphotericin B, KCZ: Ketoconazole, CBP: clinical breakpoints, ECV: epidemiological cutoff values, WT: wild type, S: susceptible, R: resistant and SDD: susceptible dose dependent.

The present results revealed a statistically significant difference in the susceptibility of *C. albicans* and NCA to AmB (P < 0.02).

### Virulence factors.

**Fig. 2:**
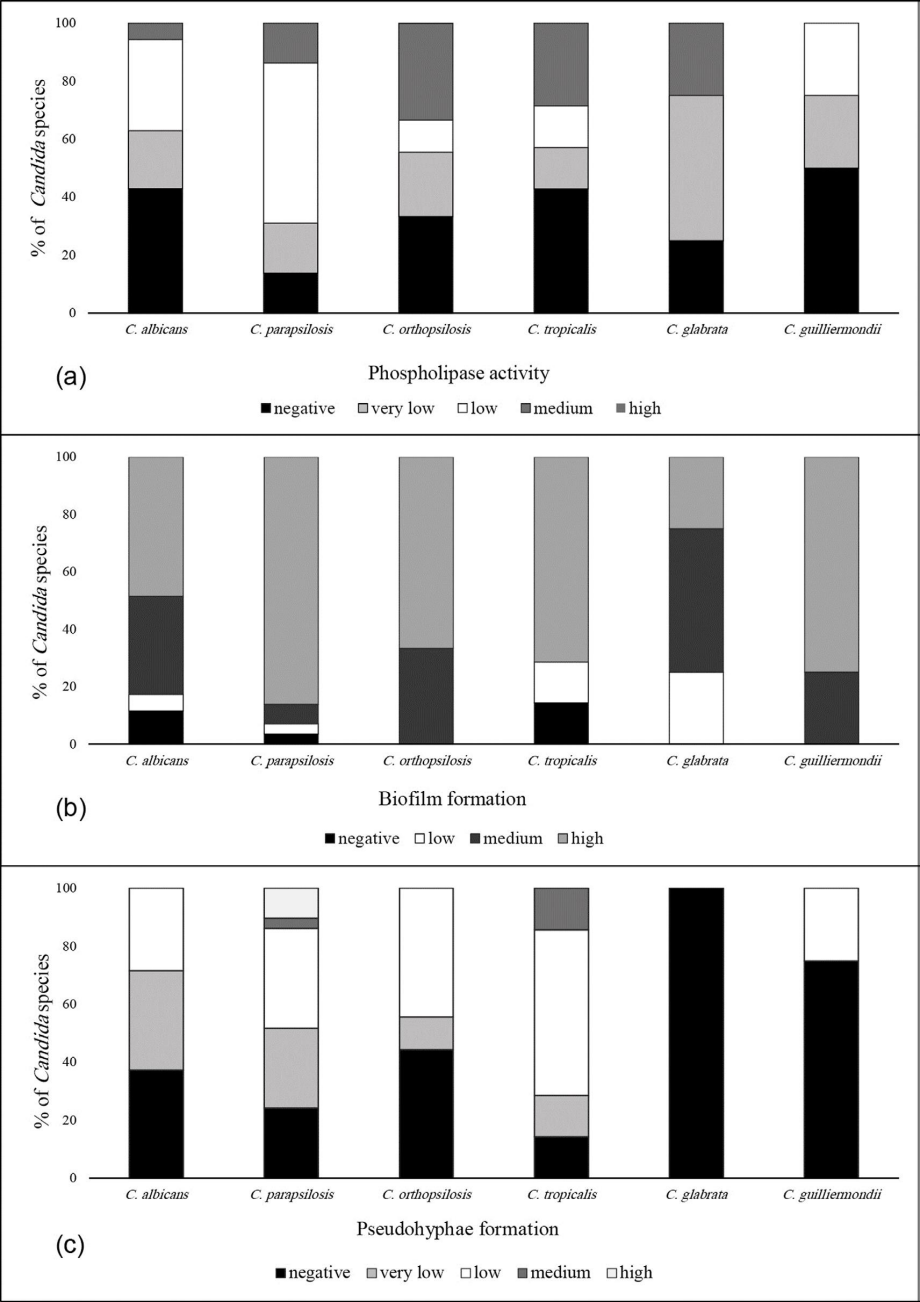
Comparison of virulence factors capacity of *Candida* species, (a): phospholipase activity; high, Pz = 0.40; medium, Pz = 0.41–0.60; low, Pz = 0.61–0.80; very low, Pz = 0.81–0.99; none, Pz = 1, (b): biofilm formation ability; low, (OD less than 0.12); medium, (OD of 0.12–0.20), or high, (OD higher than 0.20), (c): pseudohyphae construction; very low, (1–20)%; low, (30–50)%; medium, (50–80)% and high, (80–100)%.

The isolates tested exhibited various degrees of phospholipase activity (Pz value: 0.42–1). A total of 20 (57.1%) of the 35 *C. albicans* isolates and 40 (75.5%) of the 53 NCA strains exhibited phospholipase activity (P<0.05). The biofilm production was also evaluated in the 83 isolates (94.3%). Biofilm production by *C. albicans* isolates was lower than that by NCA isolates (91.4% vs. 96.2%) respectively (P<0.05). The highest phospholipase activity, biofilm formation and pseudohyphae production were seen in *C. parapsilosis* isolates (Fig. 2).

### Multilocus sequence typing (MLST).

**Fig. 3:**
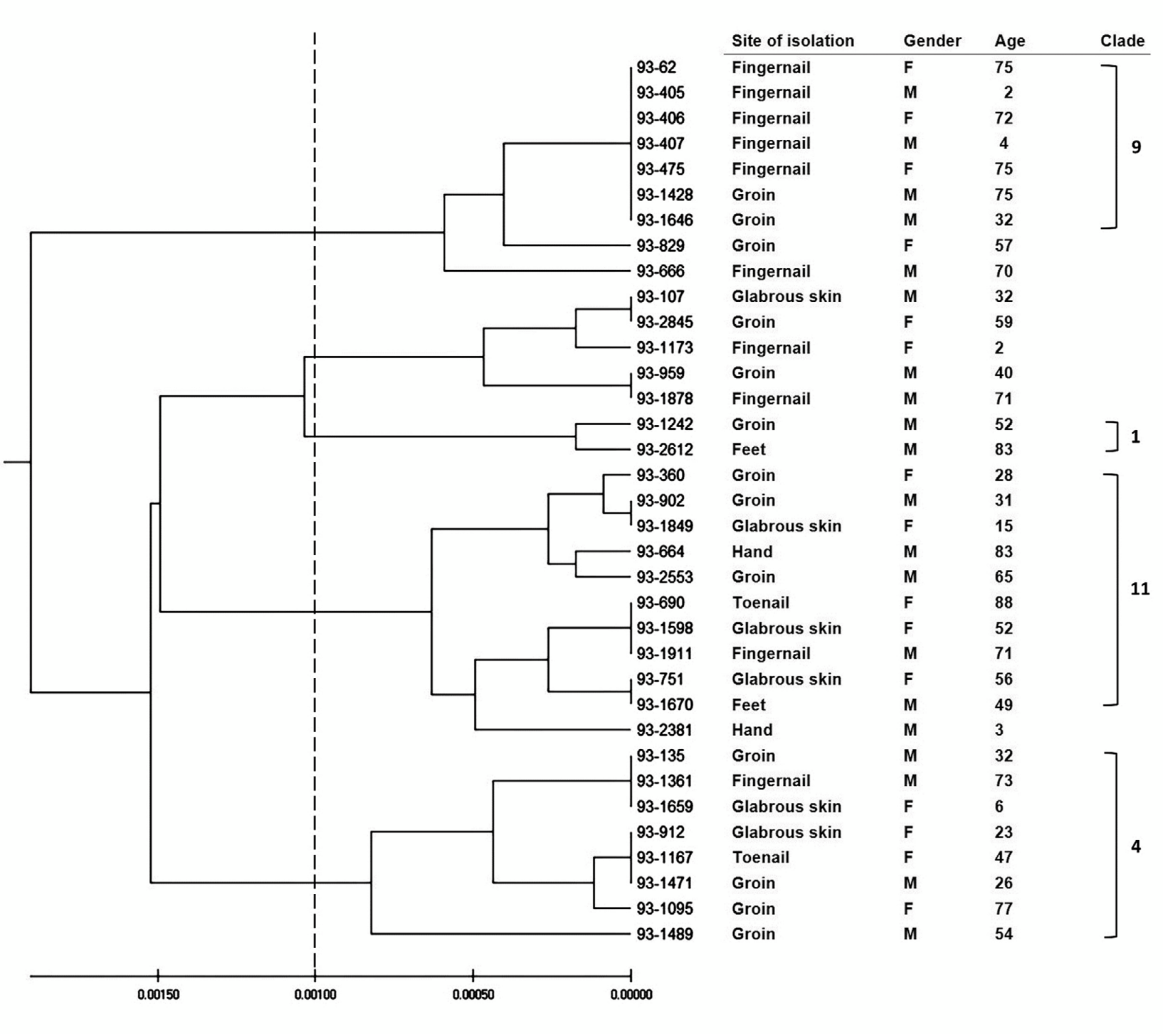
The dendrogram was constructed from UPGMA analysis based on concatenated MLST sequence of the seven loci of 35 *C. albicans* strains. Site of isolation, gender, age and clade assignments were indicated in the right columns.

The MLST data of the 35 *C. albicans* isolates consisted of 2883 nt distributed in 4 major clades (Fig. 3). Eighty eight alleles were identified in the seven loci studied. The gene VPS13 generated the highest number of alleles (n=18), while MPb1 generated the lowest number (n=6). Among the alleles, seven new alleles were determined. There were in the ADP1 locus (allelic numbers 171 and 172), VPS13 (303, 304 and 305) and ZWF1b (293 and 294). These were added to the MLST database (http://calbicans.mlst.net). The 35 isolates yielded 33 unique Diploid Sequence Type (DSTs). Seven isolates (20.0%) belonged to previously described DSTs, while 28 (80.0%) were new. Three isolates were assigned to DST 1403. Table 4 shows the allelic number for each gene and DST for all the strains included in the study. Of 35 isolates, 24 (68.5%) clustered into previously known CC, while 11 (31.5%) did not cluster in any known group. The major of isolates were grouped into three CC: 124 (20%), 461 (17.2%) and 918 (14.3%). Seventeen of the new DST clustered among already defined CCs (61%), however, 11 (39%) of the new DST could not be assigned to any known CC.

**Table 4.** Alleles, Diploid Sequence Types (DSTs) and clonal clusters for the *Candida* strains included in the study. Bold numbers are the new alleles or DST assigned by the curator of MLST database.

**Strain no.**	**AAT1a**	**ACC1**	**ADP1**	**MP1b**	**SYA1**	**VPS13**	**ZWF1b**	**DST**	**Clonal cluster**
93-62	62	3	46	3	3	215	95	3492	S
93–107	6	3	37	2	38	303	12	3493	840
93–135	8	14	8	4	34	10	8	3494	124
93–360	60	7	8	1	34	11	15	3495	461
93–405	6	3	3	2	38	10	12	3496	S
93–406	62	3	3	3	3	115	94	3497	918
93–407	62	3	3	14	3	115	95	3498	918
93–475	62	3	43	3	3	115	95	3499	918
93–664	13	3	8	4	34	20	8	3500	S
93–666	6	3	10	2	153	304	12	3501	S
93–690	60	10	3	1	34	11	15	3502	461
93–751	60	13	96	1	34	11	15	3503	461
93–829	47	14	35	28	18	213	6	3504	S
93–902	60	10	171	1	34	11	15	3505	461
93–912	8	14	8	4	50	10	8	1403	124
93–959	6	3	6	4	173	72	202	3506	S
93–1095	8	7	8	4	2	3	8	3507	124
93–1167	8	14	8	4	50	10	8	1403	124
93–1173	6	3	172	2	125	305	12	3508	S
93–1242	3	3	5	3	57	130	6	3509	367
93–1361	8	14	8	4	7	10	293	3510	124
93–1428	62	3	3	3	26	39	95	1103	918
93–1471	8	14	8	4	50	10	8	1403	344
93–1489	14	14	8	4	2	10	8	3511	124
93–1598	28	10	154	1	34	126	294	3512	S
93–1646	62	3	3	3	3	39	95	918	918
93–1659	8	14	8	4	2	3	8	481	124
93–1670	60	10	21	1	34	11	15	2216	461
93–1849	62	12	21	1	6	30	72	3513	538
93–1878	2	3	6	2	2	265	261	3514	S
93–1911	60	13	21	1	34	126	15	3515	461
93–2381	13	3	37	2	153	126	12	3516	S
93–2553	62	12	6	1	6	30	7	3517	538
93–2612	23	5	5	2	2	20	25	3518	S
93–2845	80	3	6	4	30	45	1	3519	747

S; singleton

## DISCUSSION

In the present study, cutaneous isolates of six *Candida* species (16% of cases with cutaneous candidiasis) were reported in outpatients referred to our laboratory. The prevalence of candidiasis was found to be different in various gender and age groups. In accordance with reports by Heidrich et al., women were more affected than men (25). The most frequent site of candidiasis in both genders was nail. Overall, the prevalence of cutaneous candidiasis has globally increased over the past years. The etiological pathogens and main infection sites vary according to the geographical region. It has been related that more than 90% of onychomycosis caused by yeasts, and their prevalence worldwide is up to 29% (25). This is in agreement with the results from other parts of world such as Iran by Hamedifard et al. in Tehran (26). In our study, the incidence of onychomycosis by *Candida* isolates (72.7%) were more than that report by Afshar et al. (61.9%) (27). Our results in the epidemiological alteration in distribution of *Candida* species were shown increase in prevalence of *C. albicans* and *C. parapsilosis* isolates but decrease in *C. tropicalis* and *C. guilliermondii* in comparison with the previous study in Guilan (28).

Onychomycosis was the most prevalent in the age group 50–59 years. Our results were in contrast to reports from northeast of Iran with the rate of 1.6% cutaneous candidiasis and the most prevalence of infections in the age range of 20–29 years (29).

We found some correlation among genotyping of *Candida* isolates and clinical features in phylogeny groups obtained by ITS sequencing. Whereas the cause of all of candidiasis in groins and in children was *C. albicans*, it played a minor role in onychomycosis (21.8%). All of *C. albicans* isolates were resistant to CAS. While all of the high quality of biofilm formation of *C. albicans* was shown in one clade with 31 isolates, other 4 *C. albicans* strains were medium in this ability. Each of *C. tropicalis*, *parapsilosis* and *guilliermondii* species were identified in one clade, separately. The 65.5% of *C. parapsilosis* isolates were recognized from fingernails specimens and 79% from women. Most of them were found in the age group of 50 to 60 years.

*C. tropicalis* and *C. orthopsilosis* were the causes of candidiasis in women and isolated from, fingernail lesions, exclusively. *C. tropicalis* was detected as the most common agent in the patients aged 40–50 years, whereas, *C. orthopsilosis* predominantly found in the 30–60 years age group.

In this study, the frequency of NCA species was higher (60.2%) than that of *C. albicans* (39.8%), indicating an increase in the frequency of NCA species in Iran. While the emergence of NCA species in various regions of the world is pointing towards a varied change in epidemiological behavior of the disease (30), these results in comparison with our previous reports, showed a shift from *C. albicans* as the predominant pathogen towards an increasing prevalence of the species *C. parapsilosis* (15). Similar to reports of Ghasemi et al. (31) diabetic patients had the highest susceptibility to candidiasis (28.4%).

In accordance with reports by Feng et al. (32). that *C. orthopsilosis* was isolated from cutaneous samples frequently, suggesting a possible pathogenic role of this species in superficial infections. Contrary to some reports (33), no *C. metapsilosis* strains were found in the present study.

Changes in *Candida* species distribution may influence treatment recommendations due to differences in susceptibility to antifungal agents among species. While based on the results reported by Whaley et al. (34), in the Asia-Pacific region, fluconazole resistance in *C. tropicalis* ranges from 0 to as high as 83% and the worldwide incidence of fluconazole resistance in *C. parapsilosis* disseminated infections ranges between 2 and 5%, our *C. parapsilosis* and *C. tropicalis* isolates were susceptible to fluconazole completely (P<0.04). All of our isolates were susceptible to amphotericin B, posaconazole and itraconazole (P< 0.01). Whereas the results obtained in this study corroborate previous findings in Iran (35), there was some modification in the rate of resistance to antifungals that is possibly due to the use of over the counter azole agents (13). According to susceptibility pattern of our *Candida* species obtained in this study, the best three antifungals were amphotericin B, posaconazole and fluconazole, respectively.

Recent evidence suggests that the majority of infections produced by these pathogens are associated with biological factors such as biofilm growth, phospholipase activity and pseudohyphae formation. Biofilm formation was found to occur most frequently among NCA (96.2%) than *C. albicans* (91.4%). This result was in accordance with published reports from India, Brazil and Saudi Arabia (36). There was significant difference in the biofilm forming abilities between *C. parapsilosis* and other *Candida* isolates. Phospholipase play an important role in the growth of *Candida* spp. and subsequent invasion of the host. Besides, although there has been widespread research to identify pathogenic factors in fungi, mainly in *C. albicans*, little is known about NCA isolates. Although a few studies of phospholipase activity have been undertaken for *C. tropicalis* and *C. parapsilosis*, none has been reported for other strains. Our finding was concurring with the results of previous investigations, in higher percentage of phospholipase production (53.8–74%) by *C. albicans* isolates, and in contrast to these results in NCA isolates (2–17%) (37). In the current study, the phospholipase activity of *C. albicans* was lower as compared to the NCA isolates except for *C. guilliermondii* strains. Whereas our results about higher percentage of positive phospholipase activity in NCA isolates were in accordance with Junior et al that *C. tropicalis* was reported as the highest number of positive isolates (91.7%) (38), in our finding *C. parapsilosis* was the most. Although *C. parapsilosis* demonstrated high pathogen factors capability such as pseudohyphae production, in agreement with Negri et al. (19) we did not find a clear relation between their abilities.

Molecular typing is a valuable method generally used for evaluating the genetic similarity of isolates in applied epidemiology. We used MLST to illustrate the genetic diversity and population structure of our *C. albicans* isolates through the public central database.

In our study, the profiles included five previously reported DSTs from UK by Odd et al in 2014 including 1403, 1103, 918, 481 and 2216. Three strains with the same DST (1403) were identified. Because of the greater number of isolates, the percentage of new DSTs in this study was 80.0% (28 out of 35) and higher than other studies published from Iran previously, such as the one performed by Afsarian et al. (39) reported 10 novel DSTs for *C. albicans* isolates from a burn intensive care unit in Iran. Similar to this report, CC 124 was the most frequent but its portion was decreased. Whereas, previous reports by Alastruey-Izquierdo et al. (40), showed CC 124 was the most prevalent in superficial candidiasis isolates but CC 918 was only found in candidemia strains (40), in our study, CC 124 also were the most and CC 918 the second. These differences can be due to the origin (both geographical and clinical) of the strains included in the data base. For analysis of this kind of assay, the larger group of isolates is necessary. We recognized 28 new DSTs (80%). Seventy-eight percent of our isolates clustered within 4 (i.e. 1,4, 9, 11) of the 18 known clades that fifty-eight percent of them belonged to three of five major clades (i.e. clades 1, 4, 11) as defined previously (23). Totally, 6% of isolates belong to clade 1, 29% of isolates to clade 11, 23% to clade 4 and 20% to clade 9, respectively. After 9 years, our results were differing from previous reports by Odds et al in 2007 and 2010 that clade 1 was the most prevalent one followed by clade 4, and clade 11, respectively (23). The ratio of clade 1 and 4 in our isolates were less than what reported by Afsarian et al. (2015) whereas this proportion about clade 9 was more. In comparison to previous report from Iran in 2015, clade 12 and 15 were not identified. Ge et al. (2012) found even higher percentages (79%) of new DSTs in strains isolated from superficial samples in China (40). Three new DSTs, including 3497, 3498 and 3499, assigned to CC 918, were found to be different in three loci from DSTs 918 and 1103 (Table 4). These findings may suggest micro evolutionary changes of a single strain occurring during adaptation to varying environmental conditions.

Our results showed that NCA species gained increasing importance as the etiologic agents of cutaneous *Candida* infections specially onychomycosis in Iran. Among tested antifungals, the best three antifungals were amphotericin B, posaconazole and fluconazole. As causative agents in candidiasis vary according to geographical area, treatment should be based on studies carried out regularly in the same regions through well-planned studies. Our results clearly indicate the importance of antifungal susceptibility testing as a necessity to select the drug of choice for treatment of different forms of candidiasis to overcome treatment failure and drug resistance phenomenon. We showed that NCA isolates particularly *C. parapsilosis* were more pathogenic than that of *C. albicans* isolates. According to MLST results, 28 novel sequence types with 11 singletons were reported for *C. albicans* strains with around 80% of new DSTs. Further whole genome sequencing of multiple *C. albicans* isolates from different clades, geographical locations and anatomical sources should provide further useful information regarding virulence gene family expansions and molecular epidemiology of this species.
